# kuenm: an R package for detailed development of ecological niche models using Maxent

**DOI:** 10.7717/peerj.6281

**Published:** 2019-02-06

**Authors:** Marlon E. Cobos, A. Townsend Peterson, Narayani Barve, Luis Osorio-Olvera

**Affiliations:** 1 Biodiversity Institute and Department of Ecology and Evolutionary Biology, University of Kansas, Lawrence, KS, United States of America; 2 Florida Museum of Natural History, University of Florida, Gainesville, FL, United States of America; 3 Facultad de Ciencias, Universidad Nacional Autónoma de México, Ciudad de México, México, Mexico; 4 Centro del Cambio Global y la Sustentabilidad A.C., Villahermosa, Tabasco, Mexico

**Keywords:** Extrapolation risks, Model calibration, Model projections, Model selection, Species distribution models

## Abstract

**Background:**

Ecological niche modeling is a set of analytical tools with applications in diverse disciplines, yet creating these models rigorously is now a challenging task. The calibration phase of these models is critical, but despite recent attempts at providing tools for performing this step, adequate detail is still missing. Here, we present the kuenm R package, a new set of tools for performing detailed development of ecological niche models using the platform Maxent in a reproducible way.

**Results:**

This package takes advantage of the versatility of R and Maxent to enable detailed model calibration and selection, final model creation and evaluation, and extrapolation risk analysis. Best parameters for modeling are selected considering (1) statistical significance, (2) predictive power, and (3) model complexity. For final models, we enable multiple parameter sets and model transfers, making processing simpler. Users can also evaluate extrapolation risk in model transfers via mobility-oriented parity (MOP) metric.

**Discussion:**

Use of this package allows robust processes of model calibration, facilitating creation of final models based on model significance, performance, and simplicity. Model transfers to multiple scenarios, also facilitated in this package, significantly reduce time invested in performing these tasks. Finally, efficient assessments of strict-extrapolation risks in model transfers via the MOP and MESS metrics help to prevent overinterpretation in model outcomes.

## Introduction

Ecological niche modeling (ENM) is a set of analytical tools (Peterson et al., 2011) with many potential applications in conservation planning (Franklin, 2013), climate change impacts (Searcy & Shaffer, 2016), biological invasions (Jiménez-Valverde et al., 2011), and the geography of disease transmission risk (Peterson, 2014), among others. A substantive theoretical basis did not appear until many years into the development of this field (Peterson et al., 2011)—for lack of such a conceptual framework, many models have been developed that are overly complex and that lack predictive power (Peterson & Nakazawa, 2008).

Model calibration is a process in which the aim is to determine which combination of parameters best represents the phenomenon of interest by finding the best fit with the data (Steele & Werndl, 2013). Although recent contributions to the field have highlighted effects of model settings on final results (Warren et al., 2014), parameters are still often selected based on simple protocols, and final models are generally constructed based on single parameterizations. However, multiple parameterizations can produce good fits to data, and this possibility should be considered (Spear, 1997). Identifying possible combinations of parameters may add complexity to the modeling process, but allows including critical sources of variation (Peterson, Cobos & Jiménez-García, 2018).

Performed manually, detailed model calibration and final model creation is quite time-consuming (e.g., a week or more). Hence, automating the process is essential for increasing robustness of ENMs. Recent efforts to enable model calibration have improved models (e.g., Muscarella et al., 2014), and have greatly accelerated various phases of the niche modeling process (Kass et al., 2018). However, more detailed calibration processes and automatization of more phases (e.g., model transfer, extrapolation risk analysis) are still needed.

Here, we introduce kuenm, an R package that automates important calibration and evaluation steps in ENM. In its current version, this package uses Maxent (Phillips, Anderson & Schapire, 2006) as the modeling algorithm, and automates model calibration, creation of final models and their transfers and evaluations, and assessment of extrapolation risks.

## Description and Functionality

### Processes implemented

**Figure 1:**
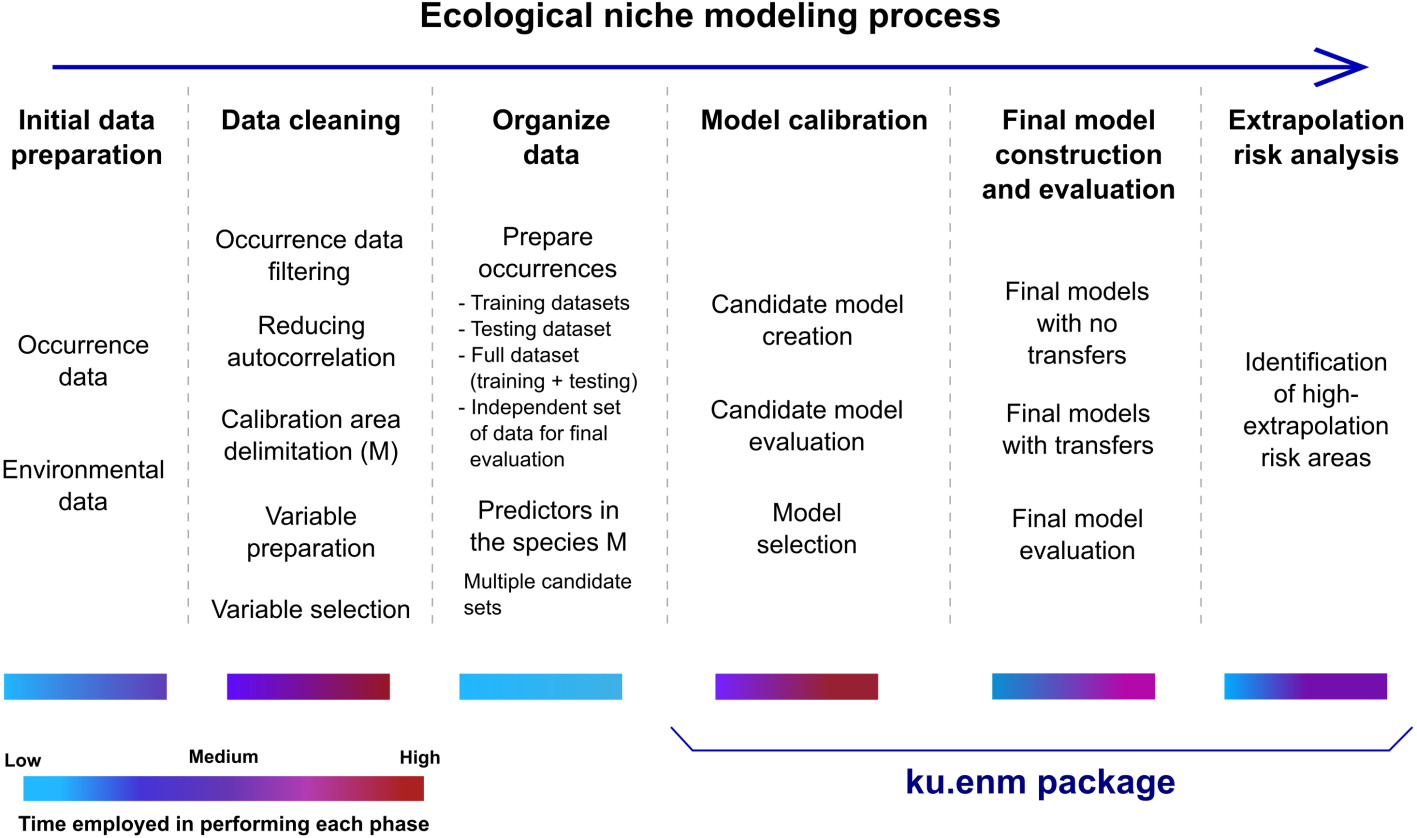
Schematic description of the ecological niche modeling process, and steps that can be performed using the kuenm package. Color bars under each step of the ecological niche modeling process reflect an approximate range of times that may be needed for execution.

This package implements three crucial phases of ENM: calibration, final model creation and evaluation, and extrapolation risk analysis (Fig. 1). Model calibration is performed in two steps: creation of large numbers of candidate models, and evaluation and selection of best models. Candidate models are created using Maxent, with different values of Maxent’s regularization multiplier parameter, combinations of feature classes, and distinct sets of environmental predictors. For each parameter setting, two models are created: one based on the complete set of occurrences, and the other based on the training data only (see data set description in Requirements and Dependencies). Model selection is based on significance, predictive ability, and complexity, in that order of priority: i.e., models are filtered first to detect those that are statistically significant; the omission rate criterion is applied to this reduced set of models; finally, among the significant and low-omission candidate models, those with values of delta AICc lower than two are selected. Significance and omission rates are calculated on models created with training data, using separate testing data subsets; model complexity is calculated on models created with the complete set of occurrences (excluding independent records, see below). We note that the full set of results of this three-part evaluation are provided, so users are able to apply their own sets of criteria.

Creation of final models in Maxent and transfers to other times or regions can be performed using the parameters selected during calibration. Final models can be created with three options of extrapolation: free extrapolation, extrapolation with clamping, and no extrapolation. Under free extrapolation settings, responses in areas environmentally different from the calibration area follow trends in the calibration environmental data. With the extrapolation and clamping setting, the response in areas with environments distinct from those in the calibration area is clamped to levels presented at the periphery of the calibration region in environmental space. Finally, under the no extrapolation setting, the response is set to zero if the environments in transfer areas are more extreme than those in areas across which the models were calibrated. Final models are evaluated based on statistical significance and omission rates using independent data (see below, in Requirements and Dependencies) when such data are available (Table 1). This evaluation performed as a post-modeling calibration process is not common enough in ENM; however, it can be useful, especially when other independent data (e.g., information on species distributions generated in explorations after creation of models) can be used to test models.

**Table 1:** Description of the main functions implemented in the kuenm R package. Additional details can be found in the main text of this manuscript and the package tutorial.

Functions	Description
kuenm_start	Generates an R Markdown file that serves as a guide to perform the main processes implemented in kuenm. This file contains a brief description of each process and chunks of code that will help beginner users in performing each of the analyses. This file can be saved in distinct formats (e.g., HTML, DOCX, and PDF) to record all the code to be used and other user comments, making the research more sharable and reproducible.
kuenm_cal	Creates Maxent candidate models. These models are created with multiple combinations of regularization multipliers, feature classes, and sets of environmental predictors. For each combination, it creates one Maxent model with the full set of occurrences, and another with training occurrence data only. Inputs are names of files and folders present in the working directory. Outputs include a folder containing all of the models and a file with Java codes for running candidate models (batch in Windows or bash in Unix), these files are written in the working directory and not stored in memory to avoid RAM limitations.
kuenm_ceval	Completes the process of calibration by evaluating candidate model performance and selecting the best ones, based on significance (partial ROC; Peterson, Papeş & Soberón, 2008), omission rates (derived from thresholded models based on *E*= user specified omission percentage; see Anderson, Lew & Peterson, 2003), and complexity (AICc; Warren, Glor & Turelli, 2010). Inputs are names of files and folders present in working directory. Outputs are written directly to the working directory, and include a file with the complete table of evaluation results, a summary of the model selection process, a table containing the evaluation metrics for only the best models, a figure of model performance across all models, and an HTML file reporting all of the results of the process to guide interpretation.
kuenm_mod	Takes the result of model evaluation and creates final models with the parameter sets selected as best. Model projections are allowed, and are called by defining the folder in which subdirectories with transfer environmental data are located; these transfers are performed automatically. Inputs are names of files and folders present in working directory. Three options of extrapolation are facilitated using this function when transfers are performed (free extrapolation, extrapolation and clamping, and no extrapolation; see Owens et al., 2013) and more than one of these options can be performed in a single run. Final models and their transfers are written directly to the working directory.
kuenm_feval	Evaluates final models based on partial ROC statistics and omission rates as assessed with independent occurrence data. Models created with the best parameter settings can be evaluated if independent data are available, to assess and evaluate their quality. Inputs are names of files and folders in the working directory; the output of this evaluation (a table with the results) is written directly to the directory.
kuenm_mmop	Calculates the mobility-oriented parity (MOP; Owens et al., 2013) metric for comparing sets of environmental conditions between the calibration area (**M**) and multiple areas or scenarios to which models are transferred (**G**). Inputs are names of files and folders in the working directory. The output maps represent the degree of similarity between conditions in **M** and **G**, wherein values of zero correspond to areas of strict extrapolation. All results are written to the working directory.
kuenm_omrat	Calculates omission rates of single models based on single or multiple threshold values (*E*; see Anderson, Lew & Peterson, 2003) specified by the user. Inputs and outputs are objects stored in memory; results indicate the rate of omission of independent occurrence data used for evaluating models created with training data.
kuenm_proc	Calculates statistical significance of single models based on the partial ROC and a threshold value (*E*; see Peterson, Papeş & Soberón, 2008) specified by the user. Inputs and outputs are objects stored in memory; outputs include a table with the partial ROC summary and the outcomes of the iterated analyses.
kuenm_mop	Calculates the MOP metric for comparisons of environmental conditions between a calibration area and a single area or scenario to which models will be transferred. Inputs and outputs are objects stored in memory; output includes a map resulting from this analysis.

Although Maxent allows assessing extrapolation via the multivariate environmental similarity surface metric (MESS; Elith, Kearney & Phillips, 2010), the mobility-oriented parity (MOP) index, implemented in kuenm is a metric proposed by Owens et al. (2013) that offers more robust measures of extrapolative conditions in final model transfers. In addition, the kuenm package allow users to use a function (kuenm_start, optional) that creates an R Markdown file that contains a brief guide to perform the main analyses implemented. This file records all user comments and lines of code used for running analyses, and can be saved in various formats, so users can share and reproduce their research easily (Table 1).

### Statistics of model performance and extrapolation risk

The statistics of model performance implemented in this package are partial ROC as a measure of statistical significance, omission rates, and AICc. Partial ROC is calculated instead of the full area under the ROC curve because the latter is not appropriate in ENM (Lobo, Jiménez-Valverde & Real, 2007; Jiménez-Valverde, 2012), and partial ROC represents a more suitable indicator of statistical significance (Peterson, Papeş & Soberón, 2008). Statistical significance is determined by a bootstrap resampling of 50% of testing data, and probabilities are assessed by direct count of the proportion of bootstrap replicates for which the AUC ratio is ≤1.0 (Peterson, Papeş & Soberón, 2008). Model evaluation, however, must go beyond significance, to measure performance as well. Performance here is measured using omission rates, which indicate how well models created with training data predict test occurrences; these rates are calculated by default at a threshold of *E* = 5% (Anderson, Lew & Peterson, 2003), but this threshold can be changed depending on user choice. Finally, to evaluate model complexity, AICc, delta AICc, and AICc weights, are calculated; AICc values indicate how well models fit to the data while penalizing complexity to favor simple models (Warren & Seifert, 2011).

Users are able to assess extrapolation risks in transfer areas with the MOP metric. The package calculates multivariate environmental distances between sites across the transfer region (**G**) and the nearest portion of the calibration region (**M** or accessible area; Soberón & Peterson, 2005) to identify regions that present situations of strict or combinational extrapolation. MOP is a metric improved for the purposes of ecological niche modeling with which to estimate extrapolation risks because it assesses environmental difference from the nearest part of the **M** region, whereas the MESS metric implemented within Maxent evaluates difference from the centroid of the **M** region in environmental space. Given the irregular nature of most environmental spaces, then, MOP is a more appropriate metric of extrapolation in niche model transfers.

### Requirements and dependencies

**Figure 2:**
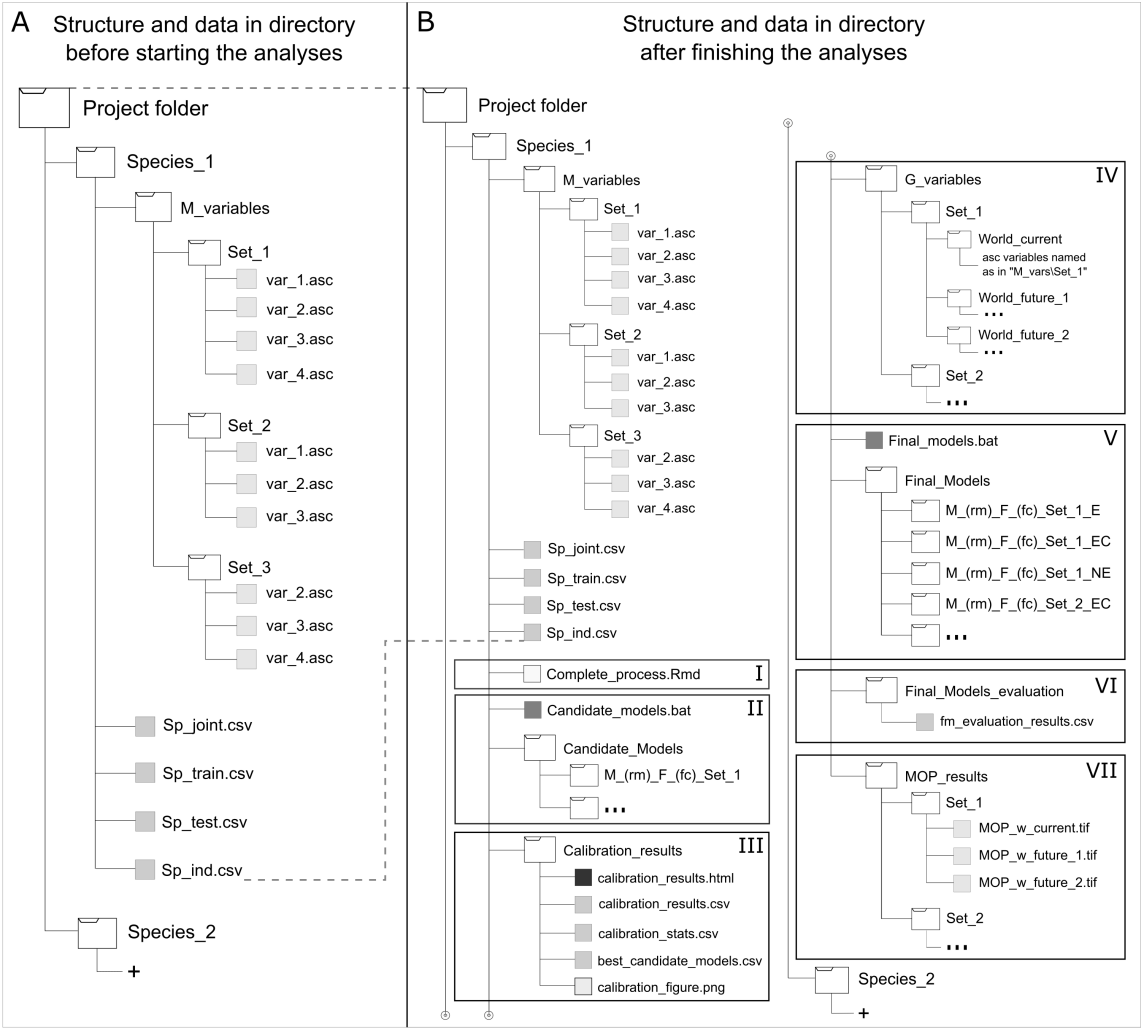
Directory structure and data for starting (A) and when finished (B) using kuenm R package functions. Roman numerals represent data needed and generated by the package: using the start function (I), creating candidate models (II), evaluating candidate models (III), preparing projection layers (IV), generating final models and its transfers (V), evaluating final models with independent data (VI), and analyzing extrapolation risks in projection areas or scenarios (VII).

To maintain simplicity and avoid memory limitations in using this package owing to the large file sizes involved in partial and final outcomes of the analyses developed by this package, a data organization structure is needed (Fig. 2). This structure allows users to run functions from a single directory per species that contains all input data needed and that is where the results will be written directly when performing model calibration, final model creation, and MOP analyses for transfer scenarios. Input data necessary to start analyses include (1) the complete set of occurrences for calibration (i.e., species occurrence records that have been filtered and thinned adequately); (2) training occurrences (part of the complete set of occurrences set aside for creating candidate models to be evaluated with testing data); (3) set of occurrences for testing candidate models (the other part of the complete set of records); and (4) one or more sets of environmental variables to be used in creating candidate models. Occurrences for training and testing models can be subsetted in multiple ways (see partition methods in Muscarella et al., 2014), but some degree of independence is desired. In addition, an entirely independent set of occurrence data (i.e., data not used during calibration that ideally come from other sources and are not spatially autocorrelated with calibration data) can be used to test final models when available. Other sets of environmental data representing distinct scenarios are required when model transfers are desired. Rtools (in Windows), Java Development Kit, and Maxent are necessary for using kuenm; R libraries imported are listed in Table S1 . Additional information and a step by step guide for using the main functions of this package can be found in its GitHub repository (https://github.com/marlonecobos/kuenm).

## Example Application

### Species and environmental data

For demonstrating the use of this package, we used as case studies the Turkey Tick, *Amblyomma americanum* (Linnaeus, 1758), and the Cuban Small-eared Toad, *Peltophryne empusa* Cope, 1862. Occurrence data were collected from online databases and the scientific literature (Alonso Bosch, 2011). As environmental predictors, we used 15 variables from the WorldClim database version 1.4 (Hijmans et al., 2005; available at http://www.worldclim.org), excluding the four that combine temperature and precipitation owing to known artifacts (Escobar et al., 2014). We clipped the environmental data layers to calibration areas defined as continental areas included within a buffer of 100 km around the tick occurrences in United States, and the entire Cuban Archipelago for the toad. Variables representing current climates and two future scenarios (representative concentration pathways; RCP 4.5 and 8.5) for the NCAR-CCSM4 general circulation model were used as transfer layers. Future data layers were obtained from the CGIAR Research Program on Climate Change, Agriculture and Food Security database (available at http://www.ccafs-climate.org/data_spatial_downscaling/). Predictors were obtained at a spatial resolution of 10′  for the tick and 30″  for the toad.

We used jackknife processes in Maxent and correlation analyses to select distinct sets of variables that contributed most to models, eliminating one variable per pair with correlations of *r* ≥ 0.8 (Table S2). We eliminated duplicates and reduced effects of spatial autocorrelation by thinning records with a distance of 50 km for the tick (final *N* = 185) and 5 km for the toad (final *N* = 67), using the spThin package (Aiello-Lammens et al., 2015) in R 3.4.4 (R Core Team, 2018). We set aside one data subset for independent model testing (7 occurrences for the tick and 3 for the toad; for demonstration purposes only) and split the remaining occurrences randomly into 50–50% (tick) and 75–25% (toad) subsets for model calibration and internal testing, respectively.

### Model calibration

For each species, we created 1,479 candidate models by combining 3 sets of environmental predictors, 17 values of regularization multiplier (0.1–1.0 at intervals of 0.1, 2–6 at intervals of 1, and 8 and 10), and all 29 possible combinations of 5 feature classes (linear = l, quadratic = q, product = p, threshold = t, and hinge = h). We evaluated candidate model performance based on significance (partial ROC, with 500 iterations and 50 percent of data for bootstrapping), omission rates (*E* = 5%), and model complexity (AICc). Best models were selected according to the following criteria: (1) significant models with (2) omission rates ≤5%. Then, from among this model set, models with delta AICc values of ≤2 were chosen as final models. Candidate model creation was performed using the **kuenm_cal** function and candidate model evaluation and best model selection was done using the **kuenm_ceval** function.

### Final models, evaluation, and extrapolation risk

We created final models for the two species using the full set of occurrences and the selected parameterizations (Table 2). We produced 10 replicates by bootstrap, with logistic outputs, and transferred these models to the world (for the tick) and all of the Cuban Archipelago (for the toad) for current and future scenarios (note that any number of scenarios can be included). Final model evaluations consisted of calculations of partial ROC and omission rates (based on *E* = 5%) using the independent dataset. Final models and their evaluations were performed with the **kuenm_mod** and **kuenm_feval** functions, respectively. When more than one best model was selected, we used the median of all replicates across parameters to consolidate results for the species. To identify extrapolation risks in model transfers, we performed MOP analyses for each species using the **kuenm_mmop** function. All analyses starting from model calibration, and the production of R Markdown files containing the codes used for running these processes (created using the **kuenm_start** function, available at https://github.com/marlonecobos/kuenm/tree/master/replicate_examples) were performed using the kuenm R package.

**Table 2:** Model performance under optimal parameters (*) and default parameters (^−^), regarding regularization multiplier (RM), feature classes (FC), and sets of predictors (Pred. Sets), for the models of the example species. Delta AICc of models with default settings are relative to the selected models. Bold numbers indicate final models that met the statistical significance and omission rate criteria during evaluation with independent data.

RM	FC	Pred. Sets	partial ROC	Omission rate 5%	AICc	Delta AICc	Weight AICc	Number of parameters
Tick								
*0.10	lqp	Set 3	**0.00**	**0.04**	3346.46	0.00	0.95	14.00
^−^1.00	lqph	Set 1	**0.00**	0.08	3385.65	39.19	0.00	41.00
^−^1.00	lqph	Set 2	**0.00**	0.08	3358.27	11.81	0.00	29.00
^−^1.00	lqph	Set 3	**0.00**	0.09	3348.13	1.67	0.00	22.00
Toad								
*0.70	p	Set 3	**0.05**	**0.00**	1508.23	0.00	0.34	3.00
*0.10	pq	Set 3	**0.03**	**0.00**	1508.39	0.16	0.98	9.00
*3.00	lqt	Set 3	**0.04**	0.00	1509.89	1.66	0.11	3.00
*4.00	lh	Set 3	**0.04**	**0.00**	1510.08	1.86	0.08	3.00
^−^1.00	lqh	Set 1	0.29	0.25	1531.90	23.67	0.00	17.00
^−^1.00	lqh	Set 2	0.29	0.25	1524.25	16.03	0.00	14.00
^−^1.00	lqh	Set 3	0.16	0.19	1530.01	21.78	0.00	14.00

### Case study outcomes

First, we explore the performance of the candidate models with respect to each of the three evaluation criteria separately. All candidate models resulted statistically significantly better than null expectations (i.e., predictions from the models coincided with testing occurrence data more frequently than would be expected by random association of points and a prediction of that areal extent) for the tick, but only 7.0% (103) were significant for the toad. Of the candidate models, 13 and 93 models met the omission rate criterion for the tick and the toad, respectively. Referring to the global minimum AICc value, for the tick, 5 models had delta AICc values ≤2, but for the toad none of the significant candidate models was close to the global minimum; note that we do not use the global minimum AICc values in selection of final models, but rather we use the minimum AICc among the significant and high-performing candidate models as our reference point.

Applying the three evaluation criteria together, for the tick, only one candidate model met the full suite of selection criteria; for the toad, however, four candidate models met the criteria (Table 2). None of the models calibrated on default settings in Maxent was selected as optimal; in fact, for the toad, none of the default-settings models was even statistically significant. After final model evaluation, the ENM for the tick and three of the four final models for the toad met both statistical significance and omission rate criteria. MOP results indicated broad areas of strict extrapolation for the tick for all transfer scenarios; for the toad, only small areas of strict extrapolation were detected in future scenarios.

Analyses took ∼10 h to process per species on a laptop computer with an i5 processor and 4GB of RAM. Note that the number of parameter combinations tested and the number of scenarios of transfer may increase or decrease processing time markedly.

## Discussion

This package allows detailed calibrations of ecological niche models in Maxent, helping to select among complex and numerous sets of parameters those that demonstrate best performance based on significance, predictive ability, and complexity level. Other options for Maxent model calibration exist (e.g., Muscarella et al., 2014); however, we introduce an alternative that allows consideration of more parameter settings (particularly different sets of environmental variables) and a more robust metric of statistical significance (i.e., partial ROC). Consideration of alternative environmental predictor variables during calibration has previously been recognized as of special importance (Peterson et al., 2011; Peterson, Cobos & Jiménez-García, 2018), yet it has not been included in model calibration and selection efforts to date. Although one could argue for including all of the environmental variables, and simply trusting in regularization and internal up- or down-weighting of variable contributions within Maxent processing, our experience indicates that such steps can lead to overfit models (Peterson, Papeş & Eaton, 2007).

**Figure 3:**
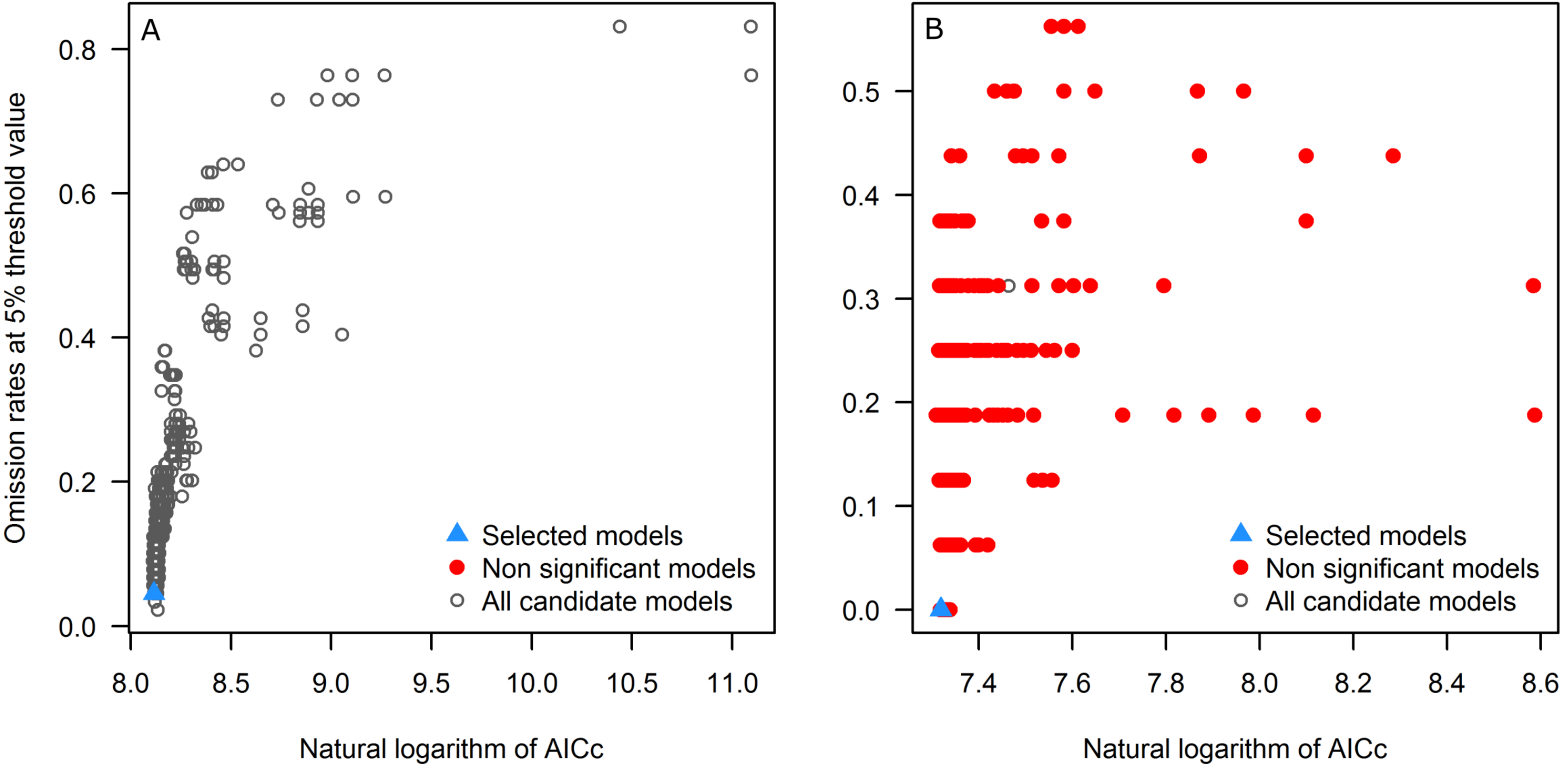
Omission rates and AICc values for all, non-significant, and selected “best” candidate models for the tick (A) and the toad (B). Models were selected based on statistical significance, omission rates, and AICc criteria.

As seen in the example applications, each species is different, and modeling ecological niches of different species will have distinct results in each phase. For instance, for the tick, all candidate models were significant, but for the toad (a Wallacean species, sensu Saupe et al., 2012: a species whose distribution is limited more by its accessible area than by the presence of limiting ecological conditions), only ∼7% were significant (Fig. 3). All candidate models created with default settings in Maxent for the toad produced non-significant models (Table 2), supporting the use of significance as a first criterion in filtering candidate models.

**Figure 4:**
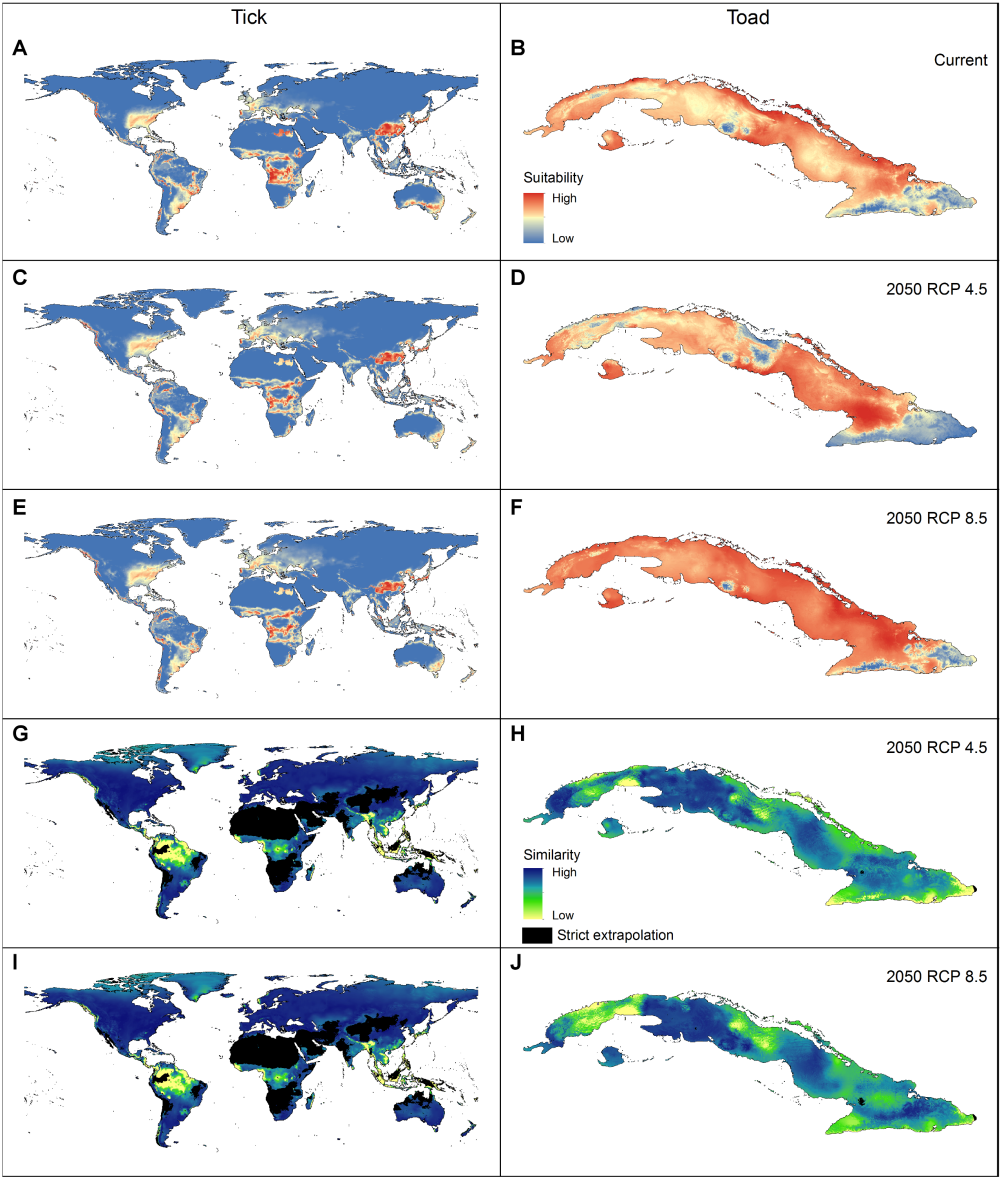
Geographic summary of the results of the analyses performed for the two example species. (A–B) Logistic output of the final models that met the selection criteria, transferred to projection areas in current and (C–F) future periods (models were produced allowing extrapolation and clamping). (G–J) Extrapolation risk in future projections (MOP results).

ENM transfers to future or past climate scenarios have become a common element in diverse analyses in biogeography and conservation (Sequeira et al., 2018). This transfer step, however, can lead to problematic extrapolations (Elith et al., 2011). Dealing with these problems is not easy, and inappropriate interpretations can be made in extrapolative areas (Figs. 4G–4H). The MOP analysis (Owens et al., 2013) is, therefore, a valuable tool for dealing with these problems by performing robust identifications of extrapolation risks.

Using this package allows users to manage model complexity in dimensions not easily manageable before, as the algorithm goes beyond software settings to include different sets of environmental predictors. Note that, depending on the selection criteria, more than one set of parameters may result in models that show best fit to the data (Table 2). Since this package allows creating suites of models with multiple sets of parameters, considering all of them in concert will improve the quality and robustness of the predictions. If more than one best model is selected, creating a consensus among those predictions will require an additional step, such as calculating the median, mean, or another metric of model agreement across parameter sets. The kuenm package differs from other similar packages in various aspects: (1) it offers a more rigorous process of model evaluation that includes partial ROC (a metric more robust than traditional ROC, and not considered in other similar packages) and automates best model selection; (2) it allows the users to test distinct sets of environmental variables, which can be used to test hypothesis of variable contribution, or to test among distinct calibration areas; (3) it automates ENM processes that have not been automated before (e.g., final model creation using multiple extrapolation options and transferring models to various scenarios at the time); and (4) it offers the chance to use the MOP metric in a context in which extrapolation risks can be analyzed for multiple scenarios to which models were transferred. These characteristics of the package make it a good option for creating more robust ENMs using Maxent considering important sources of model variation and uncertainty given by the multiple options of software settings and input data.

We used Maxent in this package in view of Maxent’s wide use within the ENM community (Merow, Smith & Silander, 2013) and its flexibility for setting parameters. Future releases will include other functions for preparing data for ENM, and for performing other post-modeling analyses; for instance, tools for assessment of model variation deriving from diverse sources (e.g., Peterson, Cobos & Jiménez-García, 2018). Although running these routines can be time-consuming, we note that model calibration steps can be similarly cumbersome in other biological optimizations, such as choosing evolutionary models for estimating phylogenetic trees (Nylander, 2004).

## Citation

Researchers using kuenm in a published paper should cite this article and indicate the used version of the package. The citation information for the current package version can be obtained using citation(“kuenm”) in R.

##  Supplemental Information

10.7717/peerj.6281/supp-1 Table S1 R packages imported, and their roles in performing multiple tasks when using kuenm

10.7717/peerj.6281/supp-2 Table S2 Candidate sets of environmental predictors tested during model calibration for the tick *Amblyomma americanum* and the toad *Peltophryne empusa.*

## Additional Information and Declarations

### Data Availability

The following information was supplied regarding data availability: Occurrence data for *Amblyomma americanum*, used in a previous analysis by  Raghavan et al. (2019), and the toad, and sets of predictors used for examples can be accessed at http://doi.org/10.17161/1808.26376. The current version of the kuenm R package can be accessed following instructions in the package short tutorial (available at GitHub: https://github.com/marlonecobos/kuenm).

## References

[ref-1] Aiello-Lammens ME, Boria RA, Radosavljevic A, Vilela B, Anderson RP (2015). spThin: an R package for spatial thinning of species occurrence records for use in ecological niche models. Ecography.

[ref-2] Alonso Bosch R (2011). Origen y diversificación del género *Peltophryne* (Amphibia: Anura: Bufonidae) en Cuba. Doctoral thesis.

[ref-3] Anderson RP, Lew D, Peterson AT (2003). Evaluating predictive models of species’ distributions: criteria for selecting optimal models. Ecological Modelling.

[ref-4] Elith J, Kearney M, Phillips S (2010). The art of modelling range-shifting species. Methods in Ecology and Evolution.

[ref-5] Elith J, Phillips SJ, Hastie T, Dudík M, Chee YE, Yates CJ (2011). A statistical explanation of MaxEnt for ecologists. Diversity and Distributions.

[ref-6] Escobar LE, Lira-Noriega A, Medina-Vogel G, Peterson AT (2014). Potential for spread of the white-nose fungus (*Pseudogymnoascus destructans*) in the Americas: use of Maxent and NicheA to assure strict model transference. Geospatial Health.

[ref-7] Franklin J (2013). Species distribution models in conservation biogeography: developments and challenges. Diversity and Distributions.

[ref-8] Hijmans RJ, Cameron SE, Parra JL, Jones PG, Jarvis A (2005). Very high resolution interpolated climate surfaces for global land areas. International Journal of Climatology.

[ref-9] Jiménez-Valverde A (2012). Insights into the area under the receiver operating characteristic curve (AUC) as a discrimination measure in species distribution modelling. Global Ecology and Biogeography.

[ref-10] Jiménez-Valverde A, Peterson AT, Soberón J, Overton JM, Aragón P, Lobo JM, Aragón P, Jiménez-Valverde A, Overton JM, Soberón J, Peterson AT (2011). Use of niche models in invasive species risk assessments. Biological Invasions.

[ref-11] Kass J, Vilela B, Aiello-Lammens M, Muscarella R, Merow C, Anderson RP (2018). Wallace: a flexible platform for reproducible modeling of species niches and distributions built for community expansion. Methods in Ecology and Evolution.

[ref-12] Lobo JM, Jiménez-Valverde A, Real R (2007). AUC: a misleading measure of the performance of predictive distribution models. Global Ecology and Biogeography.

[ref-13] Merow C, Smith MJ, Silander JA (2013). A practical guide to MaxEnt for modeling species’ distributions: what it does, and why inputs and settings matter. Ecography.

[ref-14] Muscarella R, Galante PJ, Soley-Guardia M, Boria RA, Kass JM, Uriarte M, Anderson RP (2014). ENMeval: an R package for conducting spatially independent evaluations and estimating optimal model complexity for Maxent ecological niche models. Methods in Ecology and Evolution.

[ref-15] Nylander J (2004). MrModeltest v2.

[ref-16] Owens HL, Campbell LP, Dornak LL, Saupe EE, Barve N, Soberón J, Ingenloff K, Lira-Noriega A, Hensz CM, Myers CE, Peterson AT (2013). Constraints on interpretation of ecological niche models by limited environmental ranges on calibration areas. Ecological Modelling.

[ref-17] Peterson AT (2014). Mapping disease transmission risk.

[ref-18] Peterson AT, Cobos ME, Jiménez-García D (2018). Major challenges for correlational ecological niche model projections to future climate conditions. Annals of the New York Academy of Sciences.

[ref-19] Peterson AT, Nakazawa Y (2008). Environmental data sets matter in ecological niche modelling: an example with *Solenopsis invicta* and *Solenopsis richteri*. Global Ecology and Biogeography.

[ref-20] Peterson AT, Papeş M, Eaton M (2007). Transferability and model evaluation in ecological niche modeling: a comparison of GARP and Maxent. Ecography.

[ref-21] Peterson AT, Papeş M, Soberón J (2008). Rethinking receiver operating characteristic analysis applications in ecological niche modeling. Ecological Modelling.

[ref-22] Peterson AT, Soberón J, Pearson RG, Anderson RP, Martínez-Meyer E, Nakamura M, Araújo MB (2011). Ecological niches and geographic distributions.

[ref-23] Phillips SJ, Anderson RP, Schapire RE (2006). Maximum entropy modeling of species geographic distributions. Ecological Modelling.

[ref-24] R Core Team (2018).

[ref-25] Raghavan RK, Peterson AT, Cobos ME, Ganta R, Foley D (2019). Current and future distribution of the Lone Star Tick, *Amblyomma americanum* (L.) (Acari: Ixodidae) in North America. PLOS ONE.

[ref-26] Saupe EE, Barve V, Myers CE, Soberón J, Barve N, Hensz CM, Peterson AT, Owens HL, Lira-Noriega A (2012). Variation in niche and distribution model performance: the need for a priori assessment of key causal factors. Ecological Modelling.

[ref-27] Searcy CA, Shaffer HB (2016). Do ecological niche models accurately identify climatic determinants of species ranges?. American Naturalist.

[ref-28] Sequeira AMM, Bouchet PJ, Yates KL, Mengersen K, Caley MJ (2018). Transferring biodiversity models for conservation: opportunities and challenges. Methods in Ecology and Evolution.

[ref-29] Soberón J, Peterson AT (2005). Interpretation of models of fundamental ecological niches and species’ distributional areas. Biodiversity Informatics.

[ref-30] Spear RC (1997). Large simulation models: calibration, uniqueness and goodness of fit. Environmental Modelling & Software.

[ref-31] Steele K, Werndl C (2013). Climate models, calibration, and confirmation. British Journal for the Philosophy of Science.

[ref-32] Warren DL, Glor RE, Turelli M (2010). ENMTools: a toolbox for comparative studies of environmental niche models. Ecography.

[ref-33] Warren DL, Seifert SN (2011). Ecological niche modeling in Maxent: the importance of model complexity and the performance of model selection criteria. Ecological Applications.

[ref-34] Warren DL, Wright AN, Seifert SN, Shaffer HB (2014). Incorporating model complexity and spatial sampling bias into ecological niche models of climate change risks faced by 90 California vertebrate species of concern. Diversity and Distributions.

