# Supplementary material for: kuenm: an R package for detailed development of ecological niche models using Maxent
Source: PeerJ. 2019 Feb 6;7:e6281. doi: 10.7717/peerj.6281 (PMC6368831; doi:10.7717/peerj.6281)
Supplement: Table S1 [file peerj-07-6281-s001.docx]

| Functions | Version | Functionality | Reference |
| --- | --- | --- | --- |
| dplyr | ≥0.7 | Used for performing queries during calculations of the partial ROC (functions: arrange, count, desc, group_by, full_join, mutate, reexports, and ungroup). | Wickham, H. et al. 2018. dplyr: A Grammar of Data  Manipulation. R package version 0.7.5.  https://CRAN.R-project.org/package=dplyr |
| fields | ≥9.6 | Used when calculating environmental differences between calibration and transfer areas (function rdist). | Nychka, D. et al. 2017. “fields: Tools for spatial data.” doi: 10.5065/D6W957CT. http://doi.org/10.5065/D6W957CT, R package version 9.6, www.image.ucar.edu/~nychka/Fields. |
| future | ≥1.8 | Used when performing analyses in parallel (functions: plan, multiprocess, availableCores, and sequential) | Henrik Bengtsson (2018). future: Unified Parallel and Distributed Processing in R for Everyone. R package version 1.9.0. https://CRAN.R-project.org/package=future |
| knitr | ≥1.20 | Used in concert with rmarkdown for producing html outputs, as well as for recording code and instructions for making analyses more reproducible (functions: include_graphics and kable). | Xie, Y. 2018. knitr: A General-Purpose Package for Dynamic Report Generation in R. R package version 1.20. |
| purrr |  | Used to perform functional programing in a function (function map_df) | Lionel Henry and Hadley Wickham (2018). purrr: Functional Programming Tools. R package version 0.2.5. https://CRAN.R-project.org/package=purrr |
| raster | ≥2.6 | Used in various instances for performing raster files-related tasks. Functions used: extract, freq, getValues, raster, rasterToPoints, stack, and writeRaster. | Hijmans, R. J. 2017. raster: Geographic Data Analysis and Modeling. R package version 2.6-7. https://CRAN.R-project.org/package=raster |
| Rcpp |  | Used to integrate a function written in C++ to R. | Dirk Eddelbuettel and Romain Francois (2011). Rcpp: Seamless R and C++ Integration. Journal of Statistical Software, 40(8), 1-18. URL  http://www.jstatsoft.org/v40/i08/. |
| rgdal | ≥1.2 | Required for writing GeoTiff raster files resulting from the MOP calculation. | Bivand, R. et al. 2018. rgdal: Bindings for the 'Geospatial' Data Abstraction Library. R package version 1.2-18. https://CRAN.R-project.org/package=rgdal |
| rmarkdown | ≥1.9 | Together with knitr, used for producing files that record code and general instructions for making analyses more reproducible and modular; used for producing html outputs as well (function render). | Allaire, J. J. et al. 2018. rmarkdown: Dynamic Documents for R. R package version 1.9. https://CRAN.R-project.org/package=rmarkdown |
| sp | ≥1.2 | Used for working with spatial objects (e.g., for conversions of R objects to spatial objects), functions used are coordinates and gridded. | Bivand, R. S. et al. 2013. Applied spatial data analysis with R, second edition. Springer, NY. http://www.asdar-book.org/ |
