# Supplementary material for: kuenm: an R package for detailed development of ecological niche models using Maxent
Source: PeerJ. 2019 Feb 6;7:e6281. doi: 10.7717/peerj.6281 (PMC6368831; doi:10.7717/peerj.6281)
Supplement: Table S2 [file peerj-07-6281-s002.docx]

| Environmental predictors | Tick | | |  | Toad | | |
| --- | --- | --- | --- | --- | --- | --- | --- |
|  | Set 1 | Set 2 | Set 3 |  | Set 1 | Set 2 | Set 3 |
| Annual mean temperature | x | x | x |  |  |  |  |
| Mean diurnal range | x |  |  |  |  |  |  |
| Temperature seasonality |  |  |  |  | x | x | x |
| Maximum temperature of warmest month | x | x |  |  | x |  |  |
| Minimum temperature of coldest month | x |  |  |  | x | x | x |
| Temperature annual range |  |  |  |  | x | x | x |
| Mean temperature of coldest quarter | x | x |  |  |  |  |  |
| Annual precipitation | x | x | x |  |  |  |  |
| Precipitation of wettest month | x |  |  |  | x | x | x |
| Precipitation of driest month | x | x |  |  | x | x |  |
| Precipitation seasonality | x | x | x |  |  |  |  |
| Precipitation of wettest quarter | x |  |  |  |  |  |  |
| Precipitation of driest quarter | x | x | x |  |  |  |  |
